# Quality of ChatGPT-Generated Responses to Common Patient Questions About Peripheral Nerve Stimulation: A Cross-Sectional Study

**DOI:** 10.3390/clinpract16040066

**Published:** 2026-03-25

**Authors:** Charles A. Odonkor, Muhammad Uzair Siddique, Sarvesh Palaniappan, Jacob Locklear, Sreekrishna Pokuri, Alexandra Adler, Peju Adekoya, Annie W. Hsu, Jonathan Paek, Hari Prabhakar, Yuri Chaves Martins, Christina Smith, Uzondu Osuagwu, Frederick K. Comrie, Alaa Abd El Sayed

**Affiliations:** 1 Department of Orthopaedics and Rehabilitation, Division of Physiatry, Yale New Haven Hospital, 20 York Street, New Haven, CT 06510, USA; muhammaduzair.siddique@yale.edu (M.U.S.);; 2 Department of Orthopaedics and Rehabilitation, Yale School of Medicine, Division of Physiatry, Interventional Pain Medicine, Yale University School of Medicine, 47 College Street, New Haven, CT 06511, USA; 3 Boston University Chobanian and Avedisian School of Medicine, 72 East Concord St., Boston, MA 02118, USA; 4 Department of Biology, University of North Carolina at Chapel Hill, Coker Hall, 120 South Rd, Chapel Hill, NC 27599, USA; jacob.locklear@alumni.unc.edu; 5 Department of Anesthesiology and Pain Medicine, Boston Medical Center, 725 Albany St, 7th Floor, Boston, MA 02118, USA; 6 Department of Anesthesiology and Perioperative Medicine, School of Medicine, Tufts University, 800 Washington St, Boston, MA 0211, USA; 7 Lowell General Hospital, Pain Management Center, 2 Hospital Dr, Lowell, MA 01852, USA; 8 Department of Anesthesia, Critical Care and Pain Medicine, Division of Pain Medicine, Johns Hopkins Hospital, 1800 Orleans Street, Baltimore, MD 21287, USA; 9 Houston Methodist, Department of Anesthesiology, Interventional Pain Medicine, 24518 Northwest Freeway, Suite 525, Cypress, TX 77429, USA; 10 Aviator Pain & Spine, 304 Insperon Drive, Grovetown, GA 30813, USA; 11 Department of Anesthesiology, Saint Louis University School of Medicine, Drummond Hall-2nd Floor, 3691 Rutger St., Saint Louis, MO 63110, USA; yuricmartins@gmail.com; 12 Department of Pain Medicine, Division of Anesthesiology, Critical Care, and Pain Medicine, University of Texas, MD Anderson Cancer Center, 1515 Holcombe Blvd., Houston, TX 77030, USA; 13 Department of Rehabilitation Medicine, Trinity Health of New England, 490 Blue Hills Ave, 3rd Floor Physician Suite, Hartford, CT 06112, USA; 14 Department of Anesthesiology, University of Wisconsin, Madison, Wisconsin, WI 53706, USA

**Keywords:** artificial intelligence, ChatGPT, large language models, neuromodulation, patient education, peripheral nerve stimulation

## Abstract

**Background**: Peripheral nerve stimulation (PNS) is increasingly used in selected patients with neuropathic pain, and many individuals seek supplemental online information to clarify procedural expectations and postoperative care. Large language models such as ChatGPT may provide scalable patient education; however, their performance for PNS-related questions has not been evaluated. This study assessed the reliability, accuracy, and comprehensibility of ChatGPT-5.0 responses to common PNS patient questions. **Methods**: We conducted a cross-sectional evaluation of ChatGPT-5.0 responses to 21 standardized questions derived through expert consensus, spanning pre-implantation, implantation, and post-implantation domains. Sixteen board-certified interventional pain specialists and a nurse educator independently rated each response using validated scales for reliability (1–6), accuracy (1–3), and comprehensibility (1–3). Descriptive statistics were calculated, and domain-level patterns were examined. **Results**: Clinician ratings demonstrated generally strong performance across all domains. Mean reliability was 4.7 ± 1.4, mean accuracy 2.6 ± 0.6, and mean comprehensibility 2.8 ± 0.5. Foundational questions addressing mechanisms, expectations, and postoperative care received the highest ratings. Lower ratings were observed for implantation-focused items requiring procedural nuance. No response fell below predefined acceptability thresholds, and sensitivity analyses confirmed that including one partial evaluator did not alter the observed trends. **Conclusions**: ChatGPT-5.0 generated responses to PNS-related patient questions that clinicians rated as generally reliable, accurate, and understandable, particularly for foundational and postoperative topics. Performance was more variable for procedural questions, underscoring the need for clinician oversight and verification. These findings provide a benchmark of current LLM capabilities and highlight the importance of ongoing evaluation as models evolve and as patients access versions with differing functionalities.

## 1. Introduction

Chronic neuropathic pain is a complex and heterogeneous condition that often requires multimodal, individualized treatment strategies [1]. Peripheral nerve stimulation (PNS) is one of several neuromodulation approaches used in select patients with focal neuropathic pain [2]. Its application spans conditions such as peripheral nerve injury, complex regional pain syndrome, phantom limb pain, and other refractory neuropathic syndromes. Regardless of modality, effective neuromodulation depends not only on appropriate patient selection and procedural execution, but also on clear patient understanding of mechanisms, expectations, risks, and postoperative care [3,4].

Traditional patient education in neuromodulation primarily relies on verbal counseling, supported by printed materials or online resources [5]. These approaches can vary substantially across clinical settings, may not adequately address patient-specific concerns, and are often constrained by limited time during clinical encounters. As a result, patients may seek supplemental information online, including clarification about procedural steps, postoperative expectations, or troubleshooting—areas where accuracy and clarity are essential for informed decision-making [6,7].

Large language models (LLMs), including the chat-based generative pre-trained transformer (ChatGPT), have emerged as accessible tools capable of generating conversational responses to patient questions. Early studies suggest potential utility for supporting patient education in chronic pain and interventional procedures [8]. However, LLM outputs reflect patterns learned from broad, publicly available data, and their accuracy can vary depending on the topic, terminology, and the quality of the underlying content. Because neuromodulation procedures involve device-specific workflows, safety considerations, and postoperative management steps, it cannot be assumed that LLM-generated explanations are uniformly accurate or complete [9,10,11,12,13,14].

Additionally, because LLMs evolve and different users may access different model versions or capability tiers, information quality may not be consistent across clinical, research, and patient-facing contexts. Evaluating performance at a defined point in time, therefore, provides an essential snapshot of current capabilities and limitations and may help guide the responsible incorporation of LLMs into patient education workflows. Evolving trends indicate that LLMs are increasingly integrated into everyday information-seeking behaviors. Patients frequently consult conversational AI systems to supplement clinical discussions, often asking follow-up questions related to recovery expectations, device management, or potential complications. While such tools may improve accessibility to medical information, they also introduce the possibility of incomplete or oversimplified explanations when applied to complex procedural therapies such as neuromodulation [15,16,17,18,19,20,21]. PNS involves device-specific programming parameters, peri-procedural precautions, and individualized postoperative guidance. As a result, evaluating the quality of AI-generated explanations in this domain is particularly relevant for understanding both the opportunities and the limitations of LLM-assisted patient education.

Given these considerations, no published studies have examined how ChatGPT performs when responding to common patient questions about PNS, a modality with distinct procedural logistics and device considerations compared with other neuromodulation techniques. This study evaluates the reliability, accuracy, and comprehensibility of ChatGPT-generated responses to frequently asked questions across pre-implantation, implantation, and post-implantation domains. By identifying both strengths and content gaps, these findings aim to inform best-practice strategies for incorporating AI-generated educational material into neuromodulation care.

## 2. Materials and Methods

### 2.1. Study Design

This cross-sectional study systematically analyzed clinician evaluations of ChatGPT-5.0 (OpenAI, San Francisco, CA, USA) responses to common patient questions about peripheral nerve stimulation (PNS). The methodological structure aligns with prior studies evaluating LLM performance in neuromodulation and interventional pain procedures [10,11,12,13].

### 2.2. Question Development and Content Scope

Twenty-one standardized questions were created to represent the spectrum of patient inquiry related to PNS (Table 1). Each item was derived through expert consensus among fellowship-trained interventional pain clinicians, reflecting recurring topics encountered in PNS clinical evaluation and follow-up. Question domains included mechanisms of therapy, candidate selection, procedural preparation, surgical technique, expected sensations, complication management, device programming, functional activity, troubleshooting, and long-term outcomes.

This approach parallels the methodology used in SCS, ESI, and LBP AI-education research, in which patient-facing questions were generated from clinical practice patterns, published educational materials, and documented patient priorities [10,11,12,13,14].

To enhance content validity, the study questions were designed to approximate the type of inquiries commonly encountered during clinical consultations, peri-procedural counseling, and postoperative follow-up visits. Questions were intentionally phrased using language consistent with typical patient communication rather than technical medical terminology. This approach was intended to simulate real-world patient interactions with conversational AI systems. This design strategy is consistent with prior evaluations of large language models in medical education and patient information contexts, where realistic patient-style prompts have been used to assess response quality [13,15,18]. By incorporating questions spanning the entire care pathway—from initial treatment consideration through long-term device management—the evaluation framework allowed assessment of how effectively ChatGPT communicates information across the continuum of PNS care.

### 2.3. ChatGPT Response Generation

The 21 questions were independently entered into ChatGPT-5.0. These questions addressed fundamental concepts, including PNS mechanisms, indications, procedural logistics, peri-procedural preparation, expected sensations, potential complications, device management, and long-term considerations. Using ChatGPT-5.0, each question was input to generate a detailed, patient-appropriate written response.

To reduce prompt bias, each question was submitted in uniform phrasing that emphasized clarity and patient-centered language. No additional context or follow-up prompts were provided. The model output for each question consisted of a single, complete written response, generated in a single interaction. Responses were exported directly from the interface to ensure content fidelity.

All responses were generated using ChatGPT-5.0 (OpenAI, San Francisco, CA, USA), with the same version and interface used throughout the evaluation period. Responses were recorded verbatim without modification to preserve accuracy and reproducibility.

### 2.4. Participants and Survey Structure

Sixteen evaluators participated in the study, including fifteen board-certified interventional pain physicians and one certified nurse educator with expertise in patient communication and health literacy. Fifteen participants completed ratings for all items; one participant completed only the first item. The partial dataset was included for item-level analysis of that question and excluded from aggregated analyses elsewhere. Surveys were administered via the secure Qualtrics XM survey platform (Qualtrics, Provo, UT, USA). Evaluators rated each ChatGPT-5.0 response using validated scales reflecting three primary performance constructs:

Surveys were administered via the secure Qualtrics XM platform (Qualtrics, Provo, UT, USA). Evaluators independently rated each ChatGPT-generated response using established scoring systems reflecting three constructs: reliability (1–6 scale; 1 = very poor, 6 = excellent), accuracy (1–3 scale; 1 = inaccurate, 3 = accurate), and comprehensibility (1–3 scale; 1 = difficult to understand, 3 = very clear). These scales have been used in prior neuromodulation-related LLM performance studies.

### 2.5. Data Management and Statistical Analysis

Responses and ratings were compiled and categorized by procedural phase:

- Pre-implantation (Q1–6): Mechanisms, risks, expectations;
- Implantation (Q7–13): Perioperative experience;
- Post-implantation (Q14–21): Long-term management, safety, troubleshooting.

Descriptive statistics were used because the objective was to assess overall rating patterns rather than to compare subgroups or test predefined hypotheses. All analyses were conducted using Microsoft Excel with manual verification of data entry and summary outputs.

## 3. Results

Clinician evaluations of the 21 ChatGPT-generated responses showed generally favorable ratings across all three assessment domains (Table 2). On the 6-point reliability scale, responses received a mean rating of 4.7 ± 1.4, indicating that evaluators perceived the content as adequately coherent and clinically consistent for most questions. Reliability scores ranged from 4.4 to 5.3. Question-level reliability was lowest for Q2 and Q16 (4.4 ± 1.5 and 4.4 ± 1.6, respectively) and highest for Q11 and Q21 (5.0 ± 1.4 and 5.3 ± 0.6, respectively).

Accuracy ratings on the 3-point scale yielded a mean score of 2.6 ± 0.6, reflecting clinician agreement that most responses aligned reasonably well with general PNS practices. Scores ranged from 2.4 to 2.9. Lower accuracy values were observed for Q16 and Q18 (2.4 ± 0.8 and 2.4 ± 0.9, respectively), both of which were related to postoperative considerations. Higher values were assigned to Q12 and Q21 (2.8 ± 0.6 and 2.9 ± 0.4), which addressed sensory expectations and general troubleshooting guidance.

**Figure 1:**
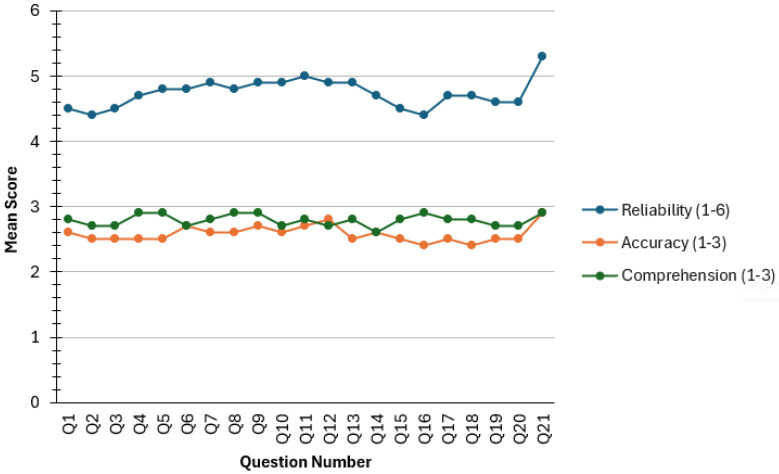
Mean reliability, accuracy, and comprehensibility scores for pre-implantation (Q1–6), implantation (Q7–13), and post-implantation (Q14–21).

Comprehensibility ratings were consistently high, with a mean of 2.8 ± 0.5 on the 3-point scale. Scores ranged from 2.6 to 2.9. Question 21 had the highest comprehensibility rating (2.9 ± 0.3), and several items (Q4, Q5, Q8, Q9, Q16) shared similarly high values (2.9 ± 0.5). The lowest comprehensibility score was noted for Q14 (2.6 ± 0.6), though still within an acceptable range for patient-facing material (Figure 1).

Domain-level patterns showed that pre-implantation and post-implantation questions tended to receive slightly higher ratings than implantation-focused items, which involve more procedural nuance. This pattern is consistent with the greater technical specificity required for perioperative questions. Including a partial dataset from one evaluator for Q1 did not materially affect the mean values or overall trends. These findings reflect clinician assessments of ChatGPT-5.0’s explanatory content and highlight areas where responses were perceived as more precise or more complete, as well as those that required greater specificity.

Inter-rater agreement was strong across all metrics, and the sensitivity analysis confirmed that including the single partial response (Q1 only) did not materially affect aggregated outcomes. Taken together, these results reflect clinician perceptions of ChatGPT-5.0’s performance, suggesting more precise and more complete responses for foundational and postoperative topics and comparatively lower clarity for items requiring procedural nuance.

## 4. Discussion

This study evaluated the quality of ChatGPT-5.0 responses to common patient questions about peripheral nerve stimulation (PNS) using clinician ratings of reliability, accuracy, and comprehensibility. Overall, raters found the responses generally coherent, clinically consistent, and understandable for foundational educational topics. These findings parallel prior work demonstrating that large language models can generate readable explanations for pain-related and interventional procedures, while also revealing performance variability in areas requiring procedural or device-specific nuance [15,16,17].

Across domains, higher ratings were observed for questions addressing mechanisms, expectations, risks, and postoperative care. These topics align more closely with the types of general medical information commonly represented in publicly available sources from which LLMs derive patterns. Conversely, lower, more variable ratings were observed for implantation-related questions [18,19]. These items require more precise detail regarding procedural steps, perioperative workflows, implant selection factors, and troubleshooting—information that is less consistently represented in general online content and therefore more challenging for models to reproduce accurately. This pattern is consistent with previous LLM evaluations in neuromodulation, minimally invasive spine procedures, and orthopedic and neurosurgical education, where accuracy tends to decline as clinical specificity increases [18,19]. Additionally, because LLMs continue to evolve and patients may rely on versions with different capabilities from those available to clinicians, real-world accuracy may vary.

The high comprehensibility ratings highlight a potential value: ChatGPT’s ability to translate complex terminology into accessible language. From a clinical perspective, this characteristic may help address a common challenge in neuromodulation practice: ensuring that patients understand complex therapeutic concepts discussed during time-limited clinical visits. Patients considering PNS must often process detailed information regarding device mechanics, expected sensory effects, activity restrictions, and long-term device management. Supplementary explanations that reinforce these concepts in clear language may therefore support patient comprehension and engagement. Prior studies examining AI-generated patient education in orthopedic surgery, spine care, and chronic pain management have similarly demonstrated that large language models can enhance readability and accessibility of medical information while maintaining generally acceptable accuracy [14,16,19].

Nevertheless, the results of this study also suggest that AI-generated explanations should not be considered a replacement for clinician counseling. Rather, LLM outputs may function best as complementary educational material that reinforces information delivered during the clinical encounter while still requiring clinician verification for accuracy and procedural specificity. Effective communication is central to informed decision-making, expectation setting, and postoperative adherence. For patients who seek supplemental explanations outside clinic visits, readable summaries may help reinforce core concepts. However, strong readability should not be interpreted as a proxy for correctness or completeness—particularly in procedural contexts where clinicians must verify nuanced or individualized decisions [15,16,17,20,21,22].

Importantly, these results represent clinician evaluations of ChatGPT-5.0 outputs at a defined point in time. Large language models evolve rapidly, and performance can differ across versions, access tiers, and user interfaces. Patients relying on freely available models may encounter a different response quality than clinicians or researchers accessing more advanced versions, a discrepancy that underscores the need for cautious interpretation. Additionally, LLM performance may change as training corpora expand, even when the application version number remains the same, or as model updates refine their ability to represent medical or device-specific information [16,19]. For these reasons, the present findings should be viewed as a snapshot of current capabilities rather than a definitive characterization of future model behavior.

This study has several strengths, including expert-designed questions, independent clinician evaluation, and analysis across multiple procedural stages. However, several limitations warrant consideration. The sample size of raters was modest and limited primarily to clinicians, which does not capture how patients interpret the material or how much they trust AI-generated guidance. In addition, the evaluation focused on static responses generated in a single interaction. In real-world use, conversational AI systems often produce iterative responses in which patients ask follow-up questions or request clarification. Such dynamic exchanges may alter the accuracy, completeness, or framing of the information provided. Future studies examining multi-turn interactions between patients and large language models may provide further insight into how these systems function in realistic educational contexts. Ratings focused solely on textual content; we did not evaluate tone, cultural tailoring, health literacy alignment, or multimodal formats (e.g., images, video, voice). Additionally, the study did not compare ChatGPT’s performance with other LLMs or with manufacturer-provided educational materials, which may offer different content scope or specificity. Future research should incorporate patient-centered evaluations, test model behavior across different versions and platforms, explore safeguards for misinformation, and examine whether AI-generated education improves patient comprehension, satisfaction, or shared decision-making in neuromodulation care.

Overall, these findings suggest that ChatGPT may serve as an adjunctive educational tool for general PNS-related questions while emphasizing the continued importance of clinician oversight. The model performed better for foundational and postoperative topics than for procedural or device-specific inquiries, highlighting areas where human verification remains essential. As LLMs continue to evolve, ongoing evaluation will be critical to ensuring that AI-generated educational content is accurate, accessible, and safely integrated into clinical practice.

## 5. Conclusions

This study evaluated ChatGPT-5.0 responses to commonly asked peripheral nerve stimulation (PNS) questions and found that clinicians rated most outputs as generally reliable, accurate, and understandable for foundational and postoperative topics. Performance was more variable for procedural and implantation-related questions, suggesting that model outputs may lack sufficient detail for patient education without clinician oversight.

These findings represent clinician assessments at a single point in time and should not be interpreted as validation of the model for clinical decision-making. Because large language models evolve rapidly—and because patients may use versions with capabilities different from those available to clinicians—ongoing evaluation is essential to ensure content accuracy, consistency, and safety. It would be instructive to evaluate how AI-generated responses influence patient comprehension, expectation alignment, and shared decision-making during neuromodulation consultations.

Within these limitations, ChatGPT may serve as an adjunctive tool to supplement general PNS education, particularly when used to reinforce broad concepts discussed in clinical encounters. Future studies incorporating patient perspectives, multiple LLM platforms, and longitudinal assessment across model updates will be necessary for determining the real-world utility and safety of AI-generated educational materials in neuromodulation care.

## Figures and Tables

**Table 1:** Common questions regarding peripheral nerve stimulation asked to ChatGPT.

Question Number	Survey Question	Domain
1	What is peripheral nerve stimulation?	Pre-implantation
2	How does PNS work?	Pre-implantation
3	What are the different PNS device options available?	Pre-implantation
4	Is PNS painful?	Pre-implantation
5	What are the risks and benefits of PNS?	Pre-implantation
6	What should I do to prepare for my procedure?	Pre-implantation
7	Where will the procedure be done?	Implantation
8	How long does the procedure take?	Implantation
9	What can I expect during and after the procedure?	Implantation
10	Will I have an incision on my back?	Implantation
11	Will I go home the same day?	Implantation
12	What does the stimulation feel like?	Implantation
13	How is the PNS device programmed?	Implantation
14	What are the different settings?	Post-implantation
15	How long will the device be on during the day?	Post-implantation
16	When can I shower after the procedure?	Post-implantation
17	When do I resume my regular medications?	Post-implantation
18	How often do I have to charge the battery?	Post-implantation
19	How soon can I return to normal activities?	Post-implantation
20	What activities should I avoid with the PNS device?	Post-implantation
21	What should I do if I experience discomfort or complications?	Post-implantation

**Table 2:** Descriptive statistics of clinician evaluations of ChatGPT-generated responses.

Question	Domain	Reliability (Mean ± SD)	Accuracy (Mean ± SD)	Comprehensibility (Mean ± SD)
Q1	Pre-Implantation	4.5 ± 1.3	2.6 ± 0.6	2.8 ± 0.6
Q2	Pre-Implantation	4.4 ± 1.5	2.5 ± 0.6	2.7 ± 0.6
Q3	Pre-Implantation	4.5 ± 1.6	2.5 ± 0.7	2.7 ± 0.6
Q4	Pre-Implantation	4.7 ± 1.4	2.5 ± 0.7	2.9 ± 0.5
Q5	Pre-Implantation	4.8 ± 1.4	2.5 ± 0.7	2.9 ± 0.5
Q6	Pre-Implantation	4.8 ± 1.4	2.7 ± 0.6	2.7 ± 0.6
Q7	Implantation	4.9 ± 1.4	2.6 ± 0.7	2.8 ± 0.6
Q8	Implantation	4.8 ± 1.5	2.6 ± 0.7	2.9 ± 0.5
Q9	Implantation	4.9 ± 1.4	2.7 ± 0.6	2.9 ± 0.5
Q10	Implantation	4.9 ± 1.4	2.6 ± 0.6	2.7 ± 0.6
Q11	Implantation	5.0 ± 1.4	2.7 ± 0.6	2.8 ± 0.6
Q12	Implantation	4.9 ± 1.4	2.8 ± 0.6	2.7 ± 0.7
Q13	Post-Implantation	4.9 ± 1.4	2.5 ± 0.7	2.8 ± 0.6
Q14	Post-Implantation	4.7 ± 1.6	2.6 ± 0.6	2.6 ± 0.6
Q15	Post-Implantation	4.5 ± 1.5	2.5 ± 0.7	2.8 ± 0.6
Q16	Post-Implantation	4.4 ± 1.6	2.4 ± 0.8	2.9 ± 0.5
Q17	Post-Implantation	4.7 ± 1.4	2.5 ± 0.7	2.8 ± 0.6
Q18	Post-Implantation	4.7 ± 1.5	2.4 ± 0.9	2.8 ± 0.6
Q19	Post-Implantation	4.6 ± 1.4	2.5 ± 0.7	2.7 ± 0.6
Q20	Post-Implantation	4.6 ± 1.6	2.5 ± 0.7	2.7 ± 0.6
Q21	Post-Implantation	5.3 ± 0.6	2.9 ± 0.4	2.9 ± 0.3

## Data Availability

The raw data supporting the conclusions of this article will be made available by the authors on request.

## Abbreviations

The following abbreviations are used in this manuscript:

AI	Artificial Intelligence
PNS	Peripheral Nerve Stimulation
LLM	Large Language Model
SCS	Spinal Cord Stimulation
